# Construction of an Acetylcholinesterase Sensor Based on Synthesized Paramagnetic Nanoparticles, a Simple Tool for Neurotoxic Compounds Assay

**DOI:** 10.3390/s17040676

**Published:** 2017-03-24

**Authors:** Adam Kostelnik, Pavel Kopel, Alexander Cegan, Miroslav Pohanka

**Affiliations:** 1 Faculty of Chemical Technology, University of Pardubice, Studentska 95, Pardubice CZ-53210, Czech Republic; st30827@student.upce.cz (A.K.); Alexander.Cegan@upce.cz (A.C.); 2 Faculty of Military Health Sciences, University of Defense, Trebesska 1575, Hradec Kralove CZ-50001, Czech Republic; 3 Department of Chemistry and Biochemistry, Mendel University in Brno, Zemedelska 1, Brno CZ-61300, Czech Republic; pavel.kopel@mendelu.cz; 4 Central European Institute of Technology, Brno University of Technology, Purkynova 123, Brno CZ-61200, Czech Republic; 5 Department of Geology and Pedology, Faculty of Forestry and Wood Technology, Mendel University in Brno, Brno CZ-61300, Czech Republic

**Keywords:** acetylcholinesterase, magnetic particles, electrochemistry, screen-printed sensor, carbofuran, galantamine, nanomaterial, nanoparticles

## Abstract

Magnetic particles (MPs) have been widely used in biological applications in recent years as a carrier for various molecules. Their big advantage is in repeated use of immobilized molecules including enzymes. Acetylcholinesterase (AChE) is an enzyme playing crucial role in neurotransmission and the enzyme is targeted by various molecules like Alzheimer’s drugs, pesticides and warfare agents. In this work, an electrochemical biosensor having AChE immobilized onto MPs and stabilized through glutaraldehyde (GA) molecule was proposed for assay of the neurotoxic compounds. The prepared nanoparticles were modified by pure AChE and they were used for the measurement anti-Alzheimer’s drug galantamine and carbamate pesticide carbofuran with limit of detection 1.5 µM and 20 nM, respectively. All measurements were carried out using screen-printed sensor with carbon working, silver reference, and carbon auxiliary electrode. Standard Ellman’s assay was used for validation measurement of both inhibitors. Part of this work was the elimination of reversible inhibitors represented by galantamine from the active site of AChE. For this purpose, we used a lower pH to get the original activity of AChE after inhibition by galantamine. We also observed decarbamylation of the AChE-carbofuran adduct. Influence of organic solvents to AChE as well as repeatability of measurement with MPs with AChE was also established.

## 1. Introduction

AChE plays a significant role in termination of signals in the cholinergic system. The mechanism of the action is based on degradation of neurotransmitter acetylcholine into non active choline and acetic acid [1]. Measuring of AChE activity is important in diagnostics or serves as a tool in analytical chemistry in inhibitor determination. A commonly acknowledged method for activity determination is Ellman’s reaction but there is potential for pH or electrochemical detection [2,3,4,5,6,7]. There is a demand for the determination of AChE inhibitors in a wide spectrum of used compounds like Alzheimer’s disease drugs (donepezil, rivastigmine, huperzine, galantamine), pesticides (carbofuran, malaoxon, malathion), and chemical warfare agents (sarin, soman, tabun, VX) [8]. MPs have been known for many years and can be prepared from many materials but most frequently iron oxides are utilized. They can be applied in a wide range of applications as can be seen from some reviews [9,10,11,12,13,14,15]. MPs have been used for many years in protein immobilization as a good platform for the attachment of proteins through free carboxyl, hydroxyl, thio, or amino groups on their surface [16,17,18]. Many protocols for enzyme immobilization on MPs have already been reported [4,19,20,21]. Silane-coupling reagents (like 3-aminopropyltrimethoxysilane or 3-aminopropyltriethoxysilane) are easily used for surface modification of synthetized MPs plus provide free amino groups which can be used for enzyme immobilization [22,23,24]. There are many different approaches proposed for the synthesis of MPs such as coprecipitation reactions, aggregation reactions, sol-gel reactions, etc. [24]. For example, maghemite nanoparticles were prepared by sodium borohydride reduction of iron chloride in ammonia solution [25,26]. The nanoparticles are easily prepared and moreover, there are hydroxo groups on their surface which are very suitable for the further modifications. The nanoparticles were used to cover Dovex for sarcosine separation as a potential prostate cancer marker. Maghemite beads modified by tetraethyl orthosilicate and 3-aminopropyl triethoxysilane can be applied for binding of H7N7 virions [27] whereas the beads covered by polyvinylpyrrolidone and gold were utilized for fluorescence resonance emission transfer (FRET)-based sarcosine detection [28].

AChE-based assays have typical lack in their inability to be used repeatedly. In this work, we focused our effort to the development of a magnetic nanoparticles based biosensor for the determination of neurotoxic compounds. We also hypothesize that the particles can be used repeatedly and lower costs in this way.

## 2. Materials and Methods

### 2.1. Materials and Equipment

Acetylcholinesterase from electric eel, lyophilized powder (≥1000 units/mg protein), acetylthiocholine chloride (ATChCl), 5,5′-dithiobis(2-nitrobenzoic acid) (DTNB), GA solution (50%), galanthamine hydrobromide, carbofuran (98%), phosphate buffer saline (PBS) pH 7.4, isopropyl alcohol (i-PrOH), dimethyl sulfoxide (DMSO), tetraethyl orthosilicate (TEOS), 3-aminopropyltriethoxysilan (APTES), *N*^1^-(3-Trimethoxysilylpropyl)diethylenetriamine (BAATMS), sodium borohydride, ammonia, iron(III) nitrate nonahydrate, sodium triphosphate, and calcium nitrate tetrahydrate were purchased from Sigma-Aldrich (St. Louis, MO, USA). Ethanol (EtOH), methanol (MeOH), sodium acetate and acetic acid were obtained from PENTA (Prague, Czech Republic). SWV assay was performed using electrochemical device PalmSens (PalmSens BV, Houten, The Netherlands) connected with computer and operated by software PSTrace 4.8.1 (PalmSens BV, Houten, The Netherlands). Screen-printed sensors (Metrohm, Herisau, Switzerland) were sized 34 × 10 × 0.5 mm with a 4 mm diameter carbon working electrode, silver reference electrode, and carbon auxiliary electrode.

### 2.2. Solutions Preparation

ATChCl solutions were prepared in concentration range from 1.25 to 20 mM. Galantamine solutions were prepared in concentration range from 25 to 100 µM. Both solutions were prepared in PBS buffer pH 7.4 and final concentration in cuvette was 10-fold less. Carbofuran inhibitor was dissolved in isopropyl alcohol in concentration range from 1.56 to 25 µM with 40-fold less concentration in cuvette. Glutaraldehyde solution (2.5%) was prepared freshly before use. All solutions were prepared into plastic microtube. For Ellman’s assay, all solutions were prepared in PBS 7.4. ATChCl solution was prepared in concentration 10 mM and DTNB in 1 mM. Concentration range of galantamine was from 25 to 100 µM and from 1.56 to 25 µM for carbofuran. Final concentrations in the cuvette were 10-fold less for ATChCl and galantamine and 40-fold less for carbofuran. Carbofuran was preincubated with AChE before substrate addition for 10 min.

### 2.3. Synthesis of Magnetic Particles

#### 2.3.1. Preparation of Maghemite

Sodium borohydride (1 g) dissolved in 3.5% ammonia (50 mL) was poured to stirred solution of iron(III) nitrate nonahydrate (7.48 g) dissolved in water (400 mL) [29]. The mixture was then heated at 100 °C for 2 h. The dark product was separated by magnet and washed several times with water. Obtained magnetic particles were combined with water to total 50 mL and 10 mL of the solution was used for every surface modification.

#### 2.3.2. Preparation of MAN-34

The maghemite was poured to i-PrOH (150 mL), 28% ammonia solution (20 mL) and TEOS (3.33 mL) were added. This mixture was stirred and heated at 40 °C overnight. The product was separated by magnet and washed several times with water and finally dried at room temperature.

#### 2.3.3. Preparation of MAN-37-NH_2_

The maghemite, i-PrOH (150 mL), 28% NH_3_ (20 mL), and TEOS (3.33 mL) were mixed, stirred and heated at 40 °C for 2 h and APTES (3.33 mL) was added and the mixture was heated for next 1 h. The product was stirred overnight at room temperature, separated by magnet and washed several times with diluted EtOH (75%). Finally, the product was left in 20 mL of EtOH (75%).

#### 2.3.4. Preparation of MAN-38-1-NH_2_

It was prepared similarly to MAN-37 but without TEOS and the product was dried after washing.

#### 2.3.5. Preparation of MAN-161-NH_2_

The maghemite suspension was diluted with MeOH (100 mL), BAATMS (0.2 mL) and 28% NH_3_ (1 mL) were mixed and stirred for 2 h. Water (50 mL) was added and stirred overnight. The product was separated by magnet and washed several times with water. Finally, the product was left in 50 mL of water.

#### 2.3.6. Preparation of MAN-164

Sodium triphosphate (0.368 g) in 50 mL of water was added to the maghemite suspension and stirred for 2 h. Then, 1 M Ca(NO_3_)_2_·4H_2_O (6 mL) was poured into the solution, continued by stirring overnight. The modified maghemite was separated by magnet, washed several times with water, and left in water (50 mL).

### 2.4. Preparation and Comparison of Particles with Bound AChE (MPs-AChE)

**Figure 1:**
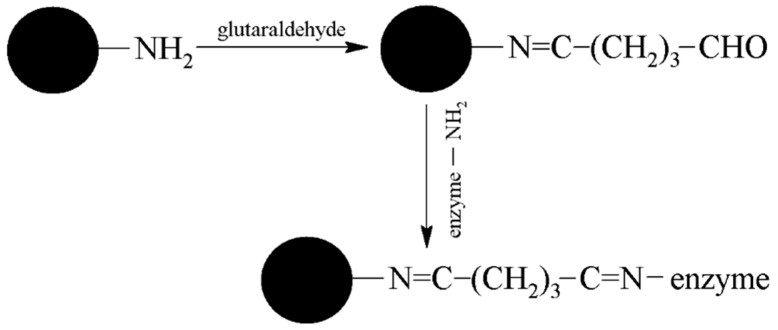
Immobilization principle of enzyme using glutaraldehyde.

MAN 34 and MAN 38-1-NH_2_ MPs were obtained as solid dust and were prepared in a concentration of 20 mg/mL for analysis. MAN 37-NH_2_, MAN 161-NH_2_, and MAN 164 MPs were suspended in solution. Before use, all MPs were well homogenized and 400 µL of each were pipetted into microtube and washed three times with 1 mL of PBS 7.4. In the next step, 400 µL of GA solution (2.5%) was added and shaken for one hour (600 rpm). After washing three times with 1 mL of PBS 7.4, 300 µL of AChE (activity for acetylthiocholine 26 U) solution was added and shaken for two hours (600 rpm). Finally, MPs-AChE were washed three times with 1 mL PBS 7.4 to remove unbound enzyme and resuspended in another 400 µL of PBS 7.4. The principle of immobilization is depicted in Figure 1. Comparison was performed using Ellman’s assay as following: 400 µL of DTNB, 450 µL of PBS 7.4, 50 µL of MPs-AChE and 100 µL of 10 mM ATChCl were mixed in standard 1.5 mL cuvette. After incubation lasting 25 min, yellow medium was separated from MPs-AChE into a clean cuvette followed by measurement of absorbance in 412 nm.

### 2.5. Electrochemical Assay

850 µL PBS 7.4, 50 µL of MPs-AChE (0.39 U per electrode), and 100 µL of 10 mM ATChCl were placed into a microtube. In the case of inhibition measurement, inhibitor was added into microtube in the appropriate concentration. For competitive inhibitors, concentration of substrate was in a concentration which did not influence competition for enzyme. After the incubation step, lasting 25 min, the reaction medium containing thiocholine was separated from MPs-AChE into clean cuvette and SWV assay was performed as described above. Setting for SWV was following: 0–1.1 V scanning range, 0.005 V potential step, 0.010 V amplitude, and frequency 1 Hz. These conditions were successfully used for thiocholine measurement in our previous work [4].

### 2.6. Validation Measurement

In a cuvette, 400 µL of Ellman’s reagent, 450 µL PBS 7.4, 50 µL of AChE, and 100 µL of 10 mM ATChCl were mixed together and an absorbance in 412 nm was measured after incubation lasting 2 min. Activity of AChE was then calculated using extinction coefficient ε = 14,150 L·mol^−1^·cm^−1^ [30].

### 2.7. Galantamine Inhibited MPs Activity Restore

Activation of MPs inhibited by galantamine was done using acetate buffer (AC) pH 5.0. MPs were incubated for 5 min and then washed three times with AC pH 5.0 followed by washing by PBS 7.4 three times.

## 3. Results and Discussion

### 3.1. Synthesis and Comparison of Magnetic Particles

**Figure 2:**
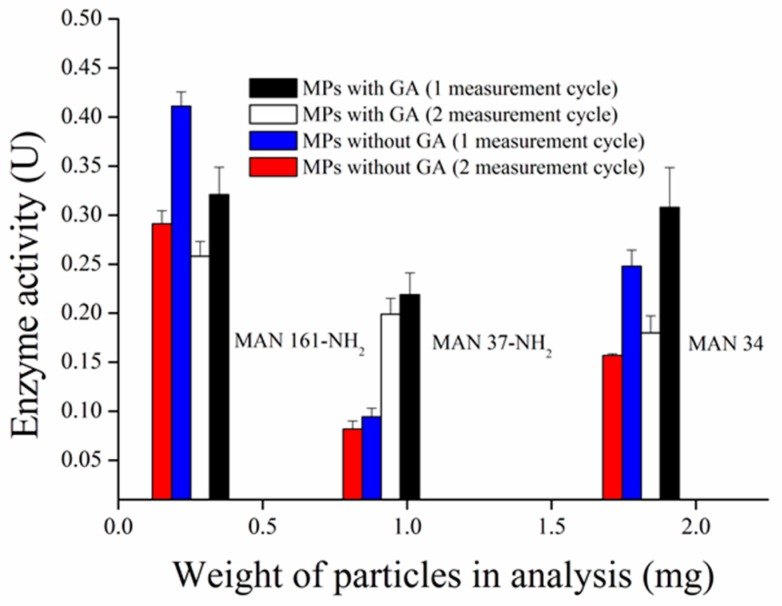
Comparision of binding process of AChE to different magnetic particles measured by Ellman’s assay. MPs with GA = magnetic particles prepared with glutaraldehyde, MPs without GA magnetic particles prepared without glutaraldehyde. Error bars indicate standard error of the mean for *n* = 3.

We have prepared five modifications of superparamagnetic particles to compare their ability to bind AChE. The surface was modified by silane (MAN-34), a combination of silane and APTES (MAN-37-NH_2_), and APTES only (MAN-38-1-NH_2_). Magnetic particles MAN-161-NH_2_ were modified with triamine, whereas MAN-164 were modified with calcium triphosphate only. Synthetized MPs were tested for ability to bound AChE with addition of bridging GA molecule and the enzyme activity was measured by Ellman’s assay. MAN 38-1-NH_2_ particles were rejected from testing because their size does not allow to form homogenous solution which is necessary for analysis. It was found that surface modification of MAN-164 particles did not allow groups for enzyme binding and simple physical adsorption do not fulfill requirement on repeated using of enzyme on their surface, therefore they were rejected for this purpose as well. Only MAN 34, MAN 37-NH_2_, and MAN 161-NH_2_ particles allow to AChE binding with/without GA molecules through either absorption in the case of MAN-34 or amino groups present on their surface. The ability of MPs to bind AChE was characterized by enzyme activity related to weight of particles took to analysis in 50 µL (Figure 2). The best results exerted MAN 161-NH_2_ particles which were able to bind the highest amount of enzyme in the smallest quantity of MPs, probably due to the high density of amino groups in BAATMS molecule per square of particle. In the case of BAATMS, the secondary amines can non-covalently interact with enzymes. This can lead to a higher affinity. Although, MAN 161-NH_2_ particles prepared without GA molecule showed bigger enzyme activity (probably due to the presence of triamine), MAN 161-NH_2_ particles prepared with GA were chosen because of higher enzyme activity in a second measuring cycle. It was probably caused by leaching of AChE from MPs prepared without GA molecule. MAN 37-NH_2_ and MAN 34 particles were able to bind AChE but did not prove to be appropriate for further measurement for smaller yields of linked enzymes. However, TEOS with APTES molecule (MAN-37-NH_2_) seems to exert weaker binding, while only the TEOS molecule (MAN-34) provides a better signal only due to non-specific interaction.

### 3.2. Substrate Measurement

**Figure 3:**
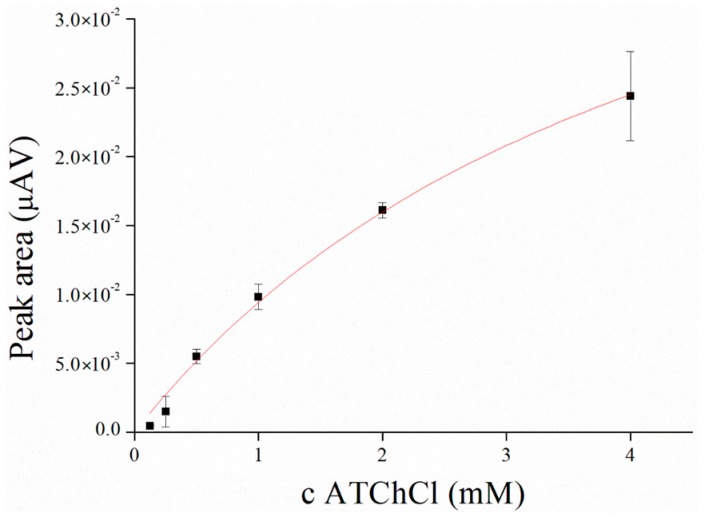
Saturation curve of AChE and acetylthiocholine as a substrate performed in PBS 7.4. Hill funciton with a coefficient of cooperativity *n* = 1 was used for fitting. Error bars indicate standard deviation for *n* = 3.

**Figure 4:**
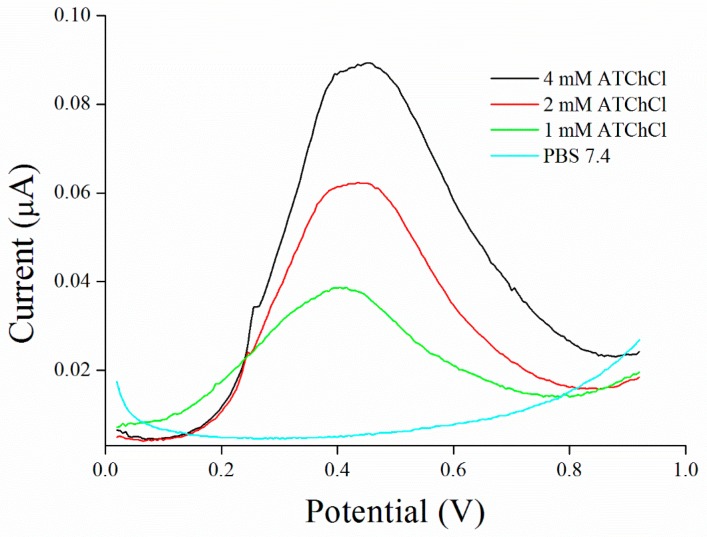
SWV curves of acetylthiocholine performed in PBS 7.4.

Saturation curve for AChE and ATChCl as a substrate was performed in concentration range from 0.125 to 4.0 mM with K_M_ value calculated to 4.56 mM compare to native enzyme, where K_M_ value was calculated to 99.57 µM (Figure 3 and Figure 4). K_M_ value seems to be strongly affected by immobilization process as was also reported by Gabrovska et al. where K_M_ value of immobilized AChE was approximately 3 mM compare to 0.9 mM of free AChE [31] or it could be influenced by external processes like irradiation as was described by Barteri et al. who calculated K_M_ value to 1.37 mM [32]. A high value of K_M_ was also provided by human mutant AChE [33,34]. Peaks forming at the potential 427 ± 22 mV were revealed during this assay.

### 3.3. Inhibitors Measurement

**Figure 5:**
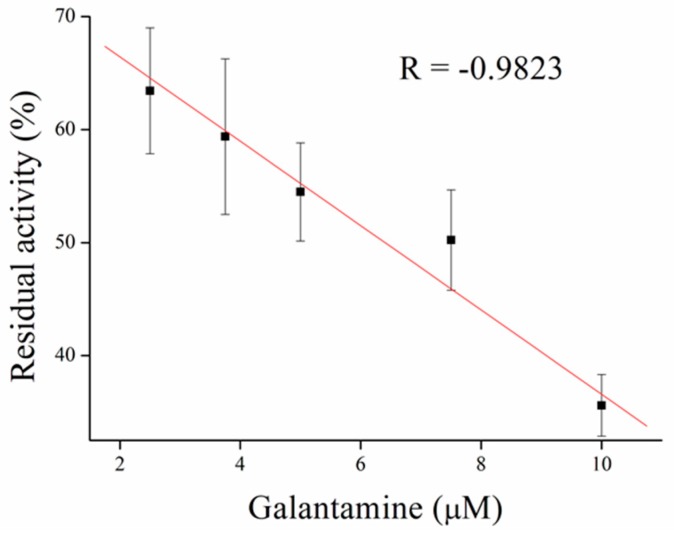
Galantamine calibration curve performed in PBS 7.4. Error bars indicate standard deviation for *n* = 3.

**Figure 6:**
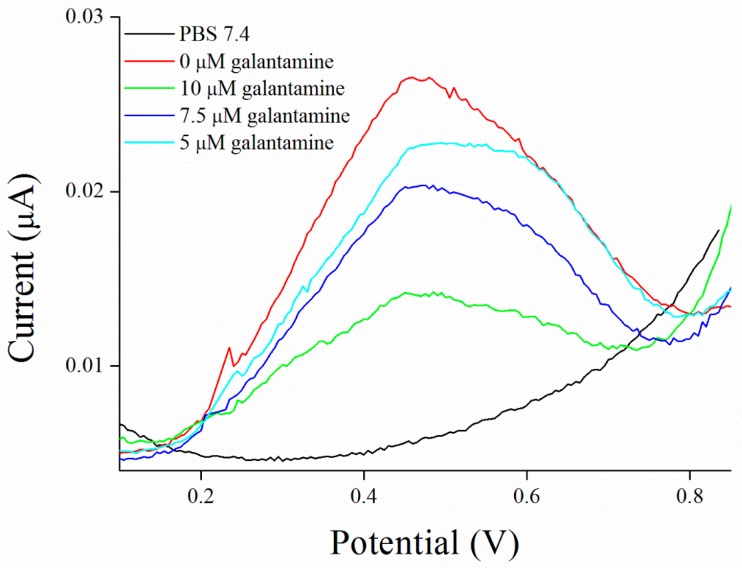
SWV curves for galantamine performed in PBS 7.4.

**Figure 7:**
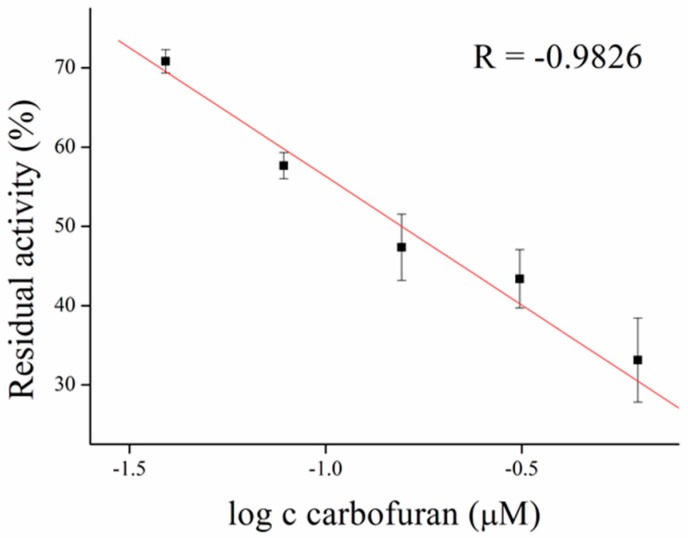
Carbofuran calibration performed in PBS 7.4. Error bars indicate standard deviation for *n* = 3.

**Figure 8:**
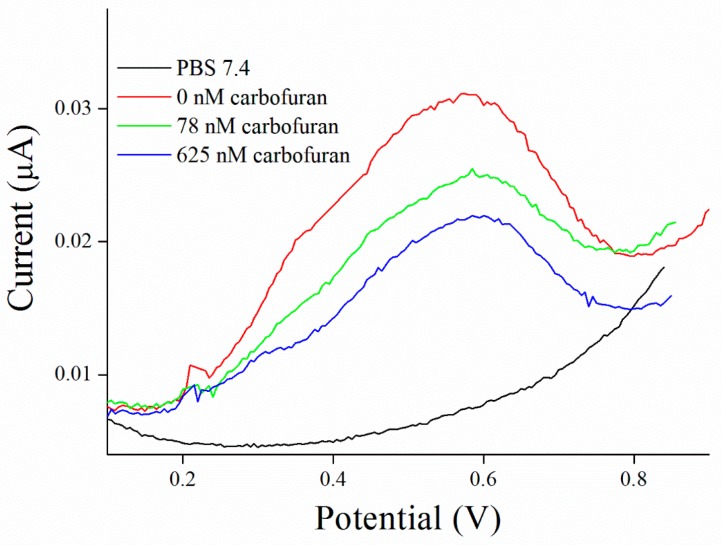
SWV curves of carbofuran performed in PBS 7.4.

**Figure 9:**
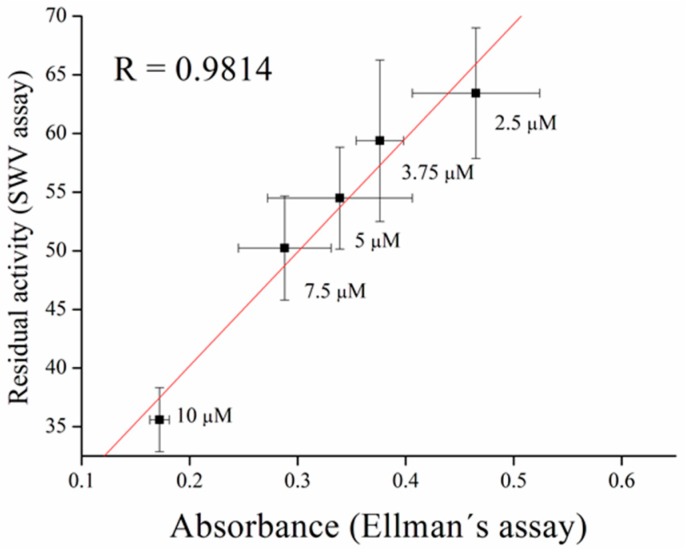
Validation of galantamine measurement compared to standard Ellman’s assay. Error bars indicate standard deviation for *n* = 3.

**Figure 10:**
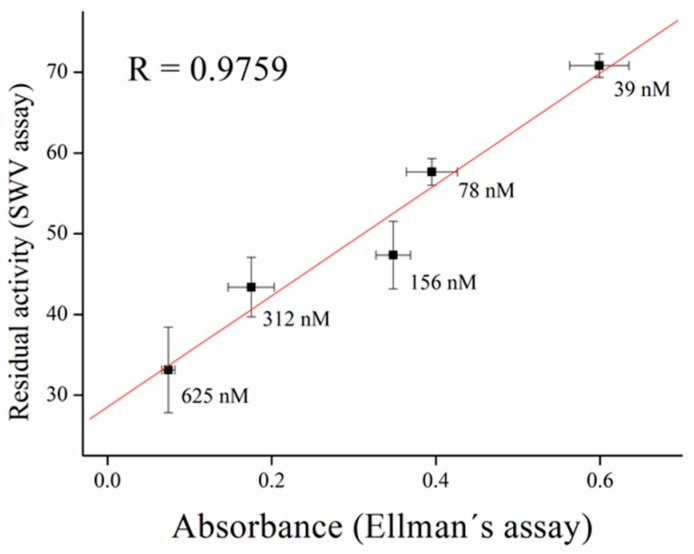
Validation of carbofuran measurement compared to standard Ellman’s assay. Error bars indicate standard deviation for *n* = 3.

Inhibitors of AChE contain wide range of substances capable reduce its activity, especially drugs used in Alzheimer’s disease treatment or pesticides in agriculture can be mentioned as the broadly available [35]. As a model molecules for our measurement, galantamine was chosen as being representative of competitive inhibitor and carbofuran was chosen as the noncompetitive inhibitor. Carbofuran as a carbamate inhibitor needs time to bind to AChE. This time was investigated while five minutes was found to be sufficient for creating this bind. Concentration of substrate for these measurements was chosen to be 1 mM of ATChCl, due to economic reasons, when thiocholine peaks were still big enough for detection. We performed calibration curves in a concentration range from 2.5 to 10 µM of galantamine (Figure 5 and Figure 6) and from 39 to 625 nM of carbofuran (Figure 7 and Figure 8) and method was validated to standard Ellman’s assay (Figure 9 and Figure 10). There are no interferences in plasma as potential real sample towards thiocholine measurement. Potential after binding of carbofuran was found to be shifted in 565 ± 20 mV. SWV assay performed only with carbofuran did not reveal any peak in the record, thus this shift is caused by interaction of carbofuran with AChE. Analysis of inhibitors is nowadays performed mainly by chromatography techniques, however, determination of AChE is a usable tool. We calculated the limit of detection for galantamine to 1.5 µM and 20 nM for carbofuran. Nevertheless, there is a drawback of this method in a relatively narrow linear range for galantamine, resulting in poor detection limit against already published methods [36,37]. On the other hand it is well established that sensitivity is decreased once the enzyme is immobilized and it is also supported by recently-achieved results [5]. Carbofuran detection limit in ppb, using AChE as recognition tool, is considered to be a good result as was previously described in literature [38,39,40].

**Figure 11:**
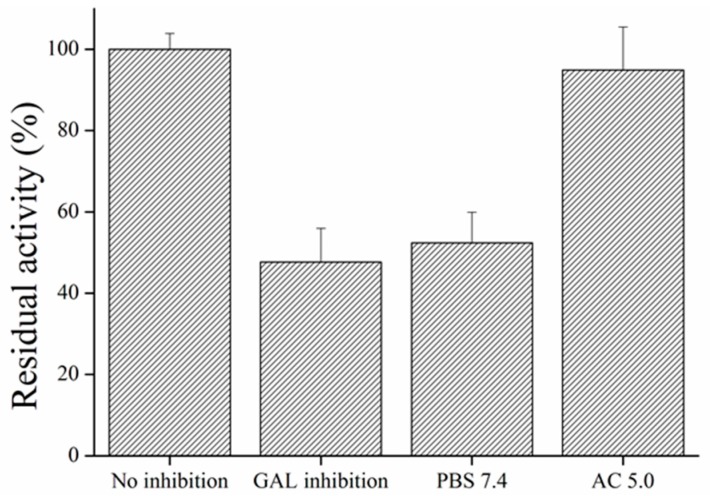
Wash out of galanthamine from galanthamine-inhibited magnetic particles. Error bars indicate standard deviation for *n* = 3.

Galantamine can inhibit enzymes as a competitive inhibitor until it is eliminated from the active site. Therefore, we washed out galantamine from AChE to get original enzyme activity. For this purpose, we tested PBS pH 7.4 and AC pH 5.0. Phosphate buffer appeared to be inapplicable for galantamine elimination when we did not wash out galantamine from the active site, while AC proved to be efficient and the enzyme appeared to be active again. The probable mechanism is in changes of charge of amino acids in the active site of the enzyme in pH 5.0 and disintegration of interaction between AChE and galantamine (Figure 11).

**Figure 12:**
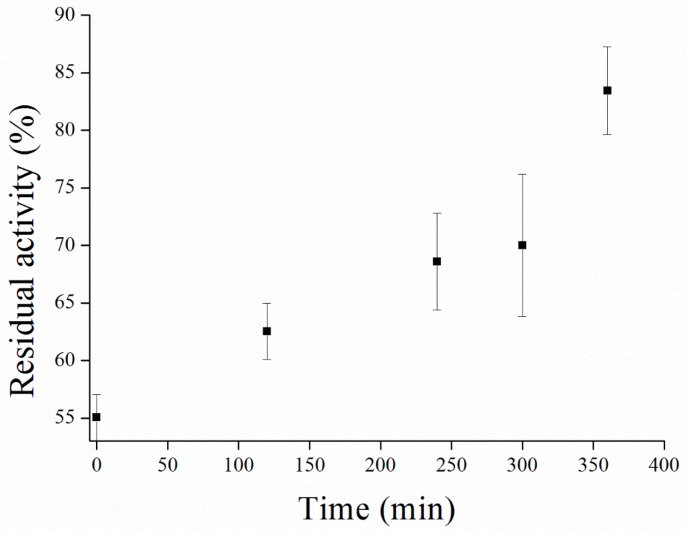
Spontaneous decarbamylation performed in PBS 7.4. Error bars indicate standard deviation for *n* = 3.

The mechanism of action of carbamate inhibitors to AChE is caused via blocking Ser residuum in the active site [41]. However, this bond undergoes spontaneous degradation over time or can be potentiated by exogenous substances [42]. Moreover, time of decarbamylation depends on length of side chain of carbamate [43]. Carbofuran adduct with BChE showed half-life about 2 h [44], while we found the half-life of the carbofuran adduct with AChE to be about 3.5 h (Figure 12).

### 3.4. Influence of Organic Solvents

**Figure 13:**
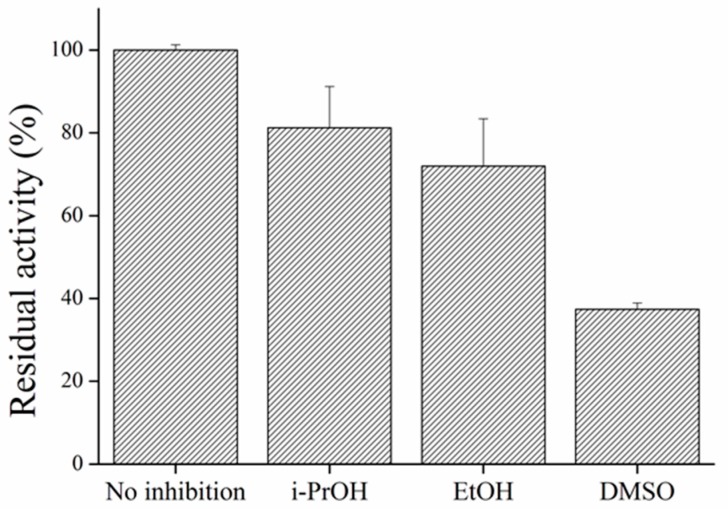
Organic solvents performed in PBS 7.4.

Organic solvents have to be considered as interference in every AChE measurement. Denaturation of enzyme molecules could have critical influence on analysis and thus it is important to keep percentage of solvents on minimum [5]. I-PrOH, EtOH, and DMSO were tested for their inhibition properties on AChE, all in 2.5% concentration. In the quoted work, DMSO was identified as a solvent capable to decrease AChE activity even in very small concentrations [45]. In tested concentrations, we found strong inhibition of AChE. Both alcohols appeared to be potent inhibitors of AChE as described earlier [46] (Figure 13).

### 3.5. Repeated Measurement with MPs-AChE

**Figure 14:**
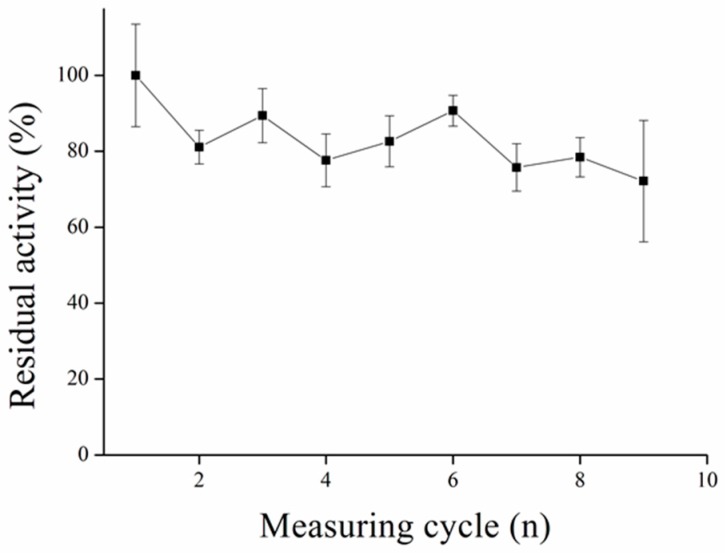
Repeatability of magnetic particles with AChE performed in PBS 7.4. Error bars indicate standard deviation for *n* = 3.

The idea of immobilization of enzymes onto MPs is in the possibility to use it repeatedly. The immobilization process via GA molecules was used before [20]. We discovered this process to be more efficient considering repeatability of measurements compared to our previous results, where immobilization to commercial MPs activated with carboxyl group and EDC reagent was used [4]. There were another immobilization protocols proposed earlier, however they require sophisticated procedures [19,47] (Figure 14). We claim that thus-modified MPs could create a good platform for automatic analyzers, microfluidics, or other uses where long stability of particles is required.

## 4. Conclusions

Synthetized MPs proved their ability to attach enzymes onto their surface via amino groups and GA molecule interaction. Compared to the immobilization process using commercial MPs and EDC reagents, prepared MPs and the use of GA appeared to be more useful when speaking about repeated measurements. We conclude that thus-prepared MPs could provide a good platform for analysis where long reagent lifetimes are needed—e.g., flow systems and so on. Inhibitors of AChE galantamine and carbofuran were measured with detection limits of 1.5 µM and 20 nM, respectively. Validation of this method was successfully performed by Ellman’s assay. Lower pH buffers were used to restore enzyme activity of inhibited particles by galantamine and spontaneous decarboxylation of the AChE-carbofuran adduct was also observed. Every analysis using AChE is influenced by organic solvents and we proved their inhibition potential in this application as well.

## References

[1] Pohanka M. (2011). Cholinesterase, a target of pharmacology and toxicology. Biomed. Pap..

[2] Pohanka M. (2015). Biosensors containing acetylcholinesterase and butyrylcholinesterase as recognition tools for detection of various compounds. Chem. Pap..

[3] Ellman G.L., Courtney K.D., Andres V., Featherstone R.M. (1961). A new and rapid colorimetric determination of acetylcholinesterase activity. Biochem. Pharmacol..

[4] Kostelnik A., Cegan A., Pohanka M. (2016). Electrochemical determination of activity of acetylcholinesterase immobilized on magnetic particles. Int. J. Electrochem. Sci..

[5] Kostelnik A., Cegan A., Pohanka M. (2016). Color change of phenol red by integrated smart phone camera as a tool for the determination of neurotoxic compounds. Sensors.

[6] Pohanka M. (2014). Voltammetric assay of butyrylcholinesterase in plasma samples and its comparison to the standard spectrophotometric test. Talanta.

[7] Pohanka M. (2013). Biosensors based on cholinesterases. Chem. Listy.

[8] Pohanka M. (2013). Cholinesterases in biorecognition and biosensors construction: A review. Anal. Lett..

[9] Lu A.H., Salabas E.L., Schuth F. (2007). Magnetic nanoparticles: Synthesis, protection, functionalization, and application. Angew. Chem. Int. Ed..

[10] Sandhu A., Handa H., Abe M. (2010). Synthesis and applications of magnetic nanoparticles for biorecognition and point of care medical diagnostics. Nanotechnology.

[11] Amstad E., Textor M., Reimhult E. (2011). Stabilization and functionalization of iron oxide nanoparticles for biomedical applications. Nanoscale.

[12] Li C.Y., Ma C., Wang F., Xi Z.J., Wang Z.F., Deng Y., He N.Y. (2012). Preparation and biomedical applications of core-shell silica/magnetic nanoparticle composites. J. Nanosci. Nanotechnol..

[13] Issa B., Obaidat I.M., Albiss B.A., Haik Y. (2013). Magnetic nanoparticles: Surface effects and properties related to biomedicine applications. Int. J. Mol. Sci..

[14] Borlido L., Azevedo A.M., Roque A.C.A., Aires-Barros M.R. (2013). Magnetic separations in biotechnology. Biotechnol. Adv..

[15] Plouffe B.D., Murthy S.K., Lewis L.H. (2015). Fundamentals and application of magnetic particles in cell isolation and enrichment: A review. Rep. Prog. Phys..

[16] Gijs M.A.M., Lacharme F., Lehmann U. (2009). Microfluidic applications of magnetic particles for biological analysis and catalysis. Chem. Rev..

[17] Centi S., Laschi S., Mascini M. (2007). Improvement of analytical performances of a disposable electrochemical immunosensor by using magnetic beads. Talanta.

[18] Liu X., Guan Y., Ma Z., Liu H. (2004). Surface modification and characterization of magnetic polymer nanospheres prepared by miniemulsion polymerization. Langmuir.

[19] Istamboulie G., Andreescu S., Marty J.-L., Noguer T. (2007). Highly sensitive detection of organophosphorus insecticides using magnetic microbeads and genetically engineered acetylcholinesterase. Biosens. Bioelectron..

[20] Günther A., Bilitewski U. (1995). Characterisation of inhibitors of acetylcholinesterase by an automated amperometric flow-injection system. Anal. Chim. Acta.

[21] Lui J., Günther A., Bilitewski U. (1997). Detection of methamidophos in vegetables using a photometric flow injection system. Environ. Monit. Assess..

[22] Yamaura M., Camilo R.L., Sampaio L.C., Macêdo M.A., Nakamura M., Toma H.E. (2004). Preparation and characterization of (3-aminopropyl)triethoxysilane-coated magnetite nanoparticles. J. Magn. Magn. Mater..

[23] Horák D., Babič M., Macková H., Beneš M.J. (2007). Preparation and properties of magnetic nano- and microsized particles for biological and environmental separations. J. Sep. Sci..

[24] Laurent S., Forge D., Port M., Roch A., Robic C., Vander Elst L., Muller R.N. (2008). Magnetic iron oxide nanoparticles: Synthesis, stabilization, vectorization, physicochemical characterizations, and biological applications. Chem. Rev..

[25] Zitka O., Cernei N., Heger Z., Matousek M., Kopel P., Kynicky J., Masarik M., Kizek R., Adam V. (2013). Microfluidic chip coupled with modified paramagnetic particles for sarcosine isolation in urine. Electrophoresis.

[26] Magro M., Sinigaglia G., Nodari L., Tucek J., Polakova K., Marusak Z., Cardillo S., Salviulo G., Russo U., Stevanato R. (2012). Charge binding of rhodamine derivative to oh- stabilized nanomaghemite: Universal nanocarrier for construction of magnetofluorescent biosensors. Acta Biomater..

[27] Heger Z., Cernei N., Guran R., Michalek P., Milosavljevic V., Kopel P., Zitka O., Kynicky J., Lany P., Adam V. (2014). Gamma-Fe_2_O_3_ magnetic core functionalized with tetraethyl orthosilicate and 3-aminopropyl triethoxysilane for an isolation of H7N7 influenza serotype virions. Int. J. Electrochem. Sci..

[28] Heger Z., Cernei N., Krizkova S., Masarik M., Kopel P., Hodek P., Zitka O., Adam V., Kizek R. (2015). Paramagnetic nanoparticles as a platform for fret-based sarcosine picomolar detection. Sci. Rep..

[29] Nejdl L., Kudr J., Cihalova K., Chudobova D., Zurek M., Zalud L., Kopecny L., Burian F., Ruttkay-Nedecky B., Krizkova S. (2014). Remote-controlled robotic platform orpheus as a new tool for detection of bacteria in the environment. Electrophoresis.

[30] Eyer P., Worek F., Kiderlen D., Sinko G., Stuglin A., Simeon-Rudolf V., Reiner E. (2003). Molar absorption coefficients for the reduced ellman reagent: Reassessment. Anal. Biochem..

[31] Gabrovska K., Marinov I., Godjevargova T., Portaccio M., Lepore M., Grano V., Diano N., Mita D.G. (2008). The influence of the support nature on the kinetics parameters, inhibition constants and reactivation of immobilized acetylcholinesterase. Int. J. Biol. Macromol..

[32] Barteri M., Pala A., Rotella S. (2005). Structural and kinetic effects of mobile phone microwaves on acetylcholinesterase activity. Biophys. Chem..

[33] Kua J., Zhang Y., Eslami A.C., Butler J.R., McCammon J.A. (2003). Studying the roles of W86, E202, and Y337 in binding of acetylcholine to acetylcholinesterase using a combined molecular dynamics and multiple docking approach. Protein Sci..

[34] Kaplan D., Barak D., Ordentlich A., Kronman C., Velan B., Shafferman A. (2004). Is aromaticity essential for trapping the catalytic histidine 447 in human acetylcholinesterase?. Biochemistry.

[35] Pohanka M., Adam V., Kizek R. (2014). Comparison of an alzheimer disease drug ability to bind acetylcholinesterase using both electrochemical and spectrophotometric assays. Res. Opin. Anim. Vet. Sci..

[36] Stoytcheva M., Zlatev R., Velkova Z., Valdez B., Ovalle M. (2009). Analytical characteristics of electrochemical biosensors. Port. Electrochim. Acta.

[37] Cuartero M., García M.S., García-Cánovas F., Ortuño J.Á. (2013). New approach for the potentiometric-enzymatic assay of reversible-competitive enzyme inhibitors. Application to acetylcholinesterase inhibitor galantamine and its determination in pharmaceuticals and human urine. Talanta.

[38] Nikolelis D.P., Simantiraki M.G., Siontorou C.G., Toth K. (2005). Flow injection analysis of carbofuran in foods using air stable lipid film based acetylcholinesterase biosensor. Anal. Chim. Acta.

[39] Pohanka M., Fusek J., Adam V., Kizek R. (2013). Carbofuran assay using gelatin based biosensor with acetylcholinesterase as a recogniton element. Int. J. Electrochem. Sci..

[40] Shulga O., Kirchhoff J.R. (2007). An acetylcholinesterase enzyme electrode stabilized by an electrodeposited gold nanoparticle layer. Electrochem. Commun..

[41] Colovic M.B., Krstic D.Z., Lazarevic-Pasti T.D., Bondzic A.M., Vasic V.M. (2013). Acetylcholinesterase inhibitors: Pharmacology and toxicology. Curr. Neuropharmacol..

[42] Kim Y.B., Jung C.H., Choi S.J., Seo W.J., Cha S.H., Sok D.E. (1992). Potentiation effect of choline esters on choline-catalysed decarbamoylation of dimethylcarbamoyl-acetylcholinesterase. Biochem. J..

[43] Wilson I.B., Harrison M.A., Ginsburg S. (1961). Carbamyl derivatives of acetylcholinesterase. J. Biol. Chem..

[44] Li H., Ricordel I., Tong L., Schopfer L.M., Baud F., Mégarbane B., Maury E., Masson P., Lockridge O. (2009). Carbofuran poisoning detected by mass spectrometry of butyrylcholinesterase adduct in human serum. J. Appl. Toxicol..

[45] Solná R., Sapelnikova S., Skládal P., Winther-Nielsen M., Carlsson C., Emnéus J., Ruzgas T. (2005). Multienzyme electrochemical array sensor for determination of phenols and pesticides. Talanta.

[46] Pohanka M., Adam V., Kizek R. (2013). An acetylcholinesterase-based chronoamperometric biosensor for fast and reliable assay of nerve agents. Sensors.

[47] Gan N., Yang X., Xie D., Wu Y., Wen W. (2010). A disposable organophosphorus pesticides enzyme biosensor based on magnetic composite nano-particles modified screen printed carbon electrode. Sensors.

